# Use of Pea Proteins in High-Moisture Meat Analogs: Physicochemical Properties of Raw Formulations and Their Texturization Using Extrusion

**DOI:** 10.3390/foods13081195

**Published:** 2024-04-14

**Authors:** Blake J. Plattner, Shan Hong, Yonghui Li, Martin J. Talavera, Hulya Dogan, Brian S. Plattner, Sajid Alavi

**Affiliations:** Department of Grain Science and Industry, Kansas State University, 201 Shellenberger Hall, Manhattan, KS 66506, USA; blakep2017@ksu.edu (B.J.P.); shanhong@ksu.edu (S.H.); yonghui@ksu.edu (Y.L.); talavera@ksu.edu (M.J.T.); dogan@ksu.edu (H.D.); bplattne@ksu.edu (B.S.P.)

**Keywords:** high-moisture meat analog, plant-based meat, extrusion, pea proteins

## Abstract

A new form of plant-based meat, known as ‘high-moisture meat analogs’ (HMMAs), is captivating the market because of its ability to mimic fresh, animal muscle meat. Utilizing pea protein in the formulation of HMMAs provides unique labeling opportunities, as peas are both “non-GMO” and low allergen. However, many of the commercial pea protein isolate (PPI) types differ in functionality, causing variation in product quality. Additionally, PPI inclusion has a major impact on final product texture. To understand the collective impact of these variables, two studies were completed. The first study compared four PPI types while the second study assessed differences in PPI inclusion amount (30–60%). Both studies were performed on a Wenger TX-52 extruder, equipped with a long-barrel cooling die. Rapid-visco analysis (RVA) and sodium dodecyl sulphate–polyacrylamide gel electrophoresis (SDS-PAGE) indicated differences in protein solubility among the different PPI types. In general, lower protein solubility led to better product quality, based on visual evaluation. Cutting strength and texture profile analysis showed increasing PPI inclusion from 30–60% led to significantly higher product hardness (14,160–16,885 g) and toughness (36,690–46,195 g. s). PPI4 led to lower product toughness (26,110 and 33,725 g. s), compared to the other PPIs (44,620–60,965 g. s). Heat gelling capacity of PPI4 was also highest among PPI types, by way of least gelation concentration (LGC) and RVA. When compared against animal meat, using more PPI (50–60%) better mimicked the overall texture and firmness of beef steak and pork chops, while less PPI better represented a softer product like chicken breast. In summary, protein content and also functionality such as cold water solubility and heat gelation dictated texturization and final product quality. High cold water solubility and poor heat gelation properties led to excessive protein cross linking and thicker yet less laminated shell or surface layer. This led to lower cutting firmness and toughness, and less than desirable product texture as compared to animal meat benchmarks. On the other hand, pea proteins with less cold water solubility and higher propensity for heat gelation led to products with more laminated surface layer, and higher cutting test and texture profile analysis response. These relationships will be useful for plant-based meat manufacturers to better tailor their products and choice of ingredients.

## 1. Introduction

Textured vegetable protein (TVP) is a food product derived from plant proteins that is experiencing rapid market growth around the world. Currently, it occupies the biggest market share among the different meat alternatives and is projected to reach over $1.5 billion by 2025 [1]. It is manufactured primarily via the extrusion process and is available in the market in several different forms [2]. The recent development of ‘high-moisture meat analogs’ (HMMAs) may be the onset of a trend away from TVP to HMMA products [3] (pp. 395–418). HMMAs are plant-based meat products designed to mimic the aesthetic and nutritional qualities of whole animal muscle meat cuts [4]. A specialized extrusion process, using a long cooling slit die, enables the production of these fresh, premium meat analogs that have the appearance and eating sensation similar to cooked animal muscle meat, while the high protein content offers a similar nutritional value [5].

Soy, wheat, and pea are the three primary plant proteins utilized in HMMA formulation. Despite the prevalence and processing advantages of soy and wheat, the meat alternative market has experienced a recent shift in demand toward clean label ingredients. Pea protein provides unique labeling opportunities, as peas are both a “non-GMO” and low allergen crop. The use of peas has been limited primarily because of their high purchasing cost and low supply, but they have become more popular [6]. The gel forming ability, solubility, and emulsifying capacity of pea protein has been found to be similar to soy protein, but its functionality is generally inferior to soy and wheat gluten [7,8]. The three primary challenges barring the widespread use of pea protein is its high cost, bitter flavor, and inferior functionality.

Prior research and industry developments have shown the potential to create single-protein HMMAs, using only pea protein [9,10,11]. In some cases, utilizing high amounts of pea protein isolate (PPI) (~80%) produces a HMMA with a thick outer shell that encompasses a soft interior center. It is predicted that diluting PPI with pea protein concentrate (PPC) can help counteract the formation of this shell and improve product quality [6,12]. Therefore, the first objective of this study was to create a high-quality, texturized HMMA product, using ascending ratios of PPI to PPC.

Differences in growing conditions in the field, combined with varying protein extraction methods utilized throughout industry has led to certain pea proteins being more functional than others [8]. This leads to processing differences, texture inconsistency, and can even alter the taste profile of the extrudates. It is important for processors to understand which commercial pea proteins will create high quality HMMA products, and identify which of their respective raw material properties most influences processability and texturization. Therefore, the second objective of this study was to determine which commercial PPI types can texturize well and create HMMAs that are comparable in quality and texture to animal meat anchors.

Thus, the overall goal of this study was to investigate the relationship between high-moisture plant-based meat analogs quality and raw material protein content and also functionality such as cold water solubility and heat gelation. It was hypothesized that these physico-chemical properties dictate texturization and final product attributes. A thorough understanding of these relationships will be useful for plant-based meat manufacturers to better tailor their products and choice of ingredients.

## 2. Materials and Methods

### 2.1. Materials and Recipes

Four pea protein isolates and one pea protein concentrate were obtained from different commercial sources or manufacturers who requested their identity not be disclosed, due to confidentiality reasons. The study focused on understanding the differences and relationships between functionality, processing and end-product quality of high-moisture meat analog products based on these primary protein sources.

The recipes were prepared, in accordance with Table 1. Recipes a had the same formulation but differed in PPI type. Isolates are denoted as PPI1, PPI2, PPI3, and PPI4, accordingly. The remaining recipes, shown in (Recipes b), used the same PPI type, i.e., PPI1, but had differing inclusion levels of PPI and PPC. For the purpose of distinguishing recipes, each recipe is denoted by PPI type and level of isolate inclusion (%). Recipes in Table 1a are denoted as PPI1-40, PPI2-40, PPI3-40, and PPI4-40. The remaining four recipes in Table 1b are denoted by PPI1-30, PPI1-40, PPI1-50, and PPI1-60.

All recipes contained equal levels of pea flour (13%) (Ingredion, Westchester, IL, USA), pea fiber (5%) (Cosucra, Warcoing, Pecq, Belgium), granulated salt (2%) (Cargill, Wayzata, MN, USA), and high oleic sunflower oil (2%) (Columbus Vegetable Oils, Des Plains, IL, USA). Ingredients were mixed together for each recipe in 150 lb batches. Protein content was measured for each PPI and PPC. Results were as follows: PPI1 = 80%, PPI2 = 77.19%, PPI3 = 78.75%, PPI4 = 76.88%, PPC = 53.22%. The slight difference in protein content between various pea protein isolates was most probably due to different methods of isolation employed by the manufacturers. Differences in amino acids were not expected as all of PPIs were based on yellow peas. The nutritional composition of the remaining ingredients was estimated from the specifications provided by the suppliers. Table 2 shows the overall nutritional composition of each recipe.

For texture comparison of HMMAs to animal muscle meat products, beef steak (USDA choice beef top sirloin steak boneless), pork chops (pork loin center cut chops boneless), chicken breast (Tyson boneless skinless chicken breasts), and salmon fillets (salmon Atlantic fresh farm raised fillet family pack color added) were purchased from Dillons Food Store (Manhattan, KS, USA) and kept refrigerated until use.

### 2.2. Moisture and Protein Contents

Moisture content of the PPIs, PPC, and raw recipes was measured according to AACC 44-19.01 [13]. Approximately 2 g of each sample was dried at 135 °C for 2 h. Analysis was completed in triplicate.

The protein content of each PPI and PPC was analyzed, according to AACC method 46-30.01 [14], using a LECO analyzer. A nitrogen to protein conversion factor of 6.25 was used and results were reported on an as-is basis. Samples were tested in triplicate.

### 2.3. Particle Size

Particle size of the raw PPIs and PPC was analyzed using an Alpine Air Jet Sieve E200 LS (Hosokawa Alpine Group, Augsburg, Germany). 100 g of protein sample was weighed and loaded onto finest mesh screen. A negative pressure of 3400 Pa was applied to the underside of the sieve. Meanwhile, a rotating arm spun counterclockwise to disperse airflow and fluidize material sitting on the screen. This combination of pressure and dispersion removes and transports particles finer than the screen, through the sieve, and into a collecting jar. Nine sieves were used, starting at the finest mesh size and progressively increasing to the largest mesh size. The set of sieves used are as follows: 25, 32, 53, 63, 75, 90, 106, 125, and 250 μm. Times for sieving for each respective sieve are as follows: 4, 3, 3, 3, 3, 3, 3, 3, 2 min. Weights of the overs were recorded and transferred to the next consecutive sieve. Cumulative distribution was calculated following analysis, based on recorded weights. Analysis was completed in duplicate.

### 2.4. Protein Solubility

Protein solubility for each PPI and PPC was determined according to the method reported by Shen et al., (2021) [15]. 0.4 g of protein was dispersed in 10 mL of water to attain 4% (*w*/*v*) solution. The suspensions were adjusted to pH 3 to 11 using either 1 M HCl or NaOH, accordingly. Each suspension was stirred for 30 min at room temperature, followed by centrifugation at 4000× *g* for 30 min to remove insoluble residues. Protein content in supernatants was determined using the Biuret method with BSA as a standard, with analysis being performed using a double beam spectrophotometer (VWR UV-6300PC), at 540 nm absorbance. Total protein content of original samples was measured by dissolving in DI water and adjusting to pH 13. Protein solubility was calculated using the following equation:

(1) $$\mathrm{Protein}\;\mathrm{Solubility}\;\left(\%\right)=\frac{\mathrm{Protein}\;\mathrm{content}\;\mathrm{in}\;\mathrm{the}\;\mathrm{supernatant}}{\mathrm{Total}\;\mathrm{protein}\;\mathrm{content}\;\mathrm{in}\;\mathrm{the}\;\mathrm{original}\;\mathrm{sample}}$$

### 2.5. Sodium Dodecyl Sulphate-Polyacrylamide Gel Electrophoresis (SDS-PAGE)

SDS-PAGE was performed following the method of Laemmli (1970) [16], under reducing conditions, to determine the protein molecular weight distributions of the PPIs and PPC. Protein samples were diluted with deionized water to 0.015% concentration and then centrifuged at 8000× *g* force for 5 min. The samples were then suspended in treatment buffer, consisting of 277.8 mM Tris-HCl (pH 6.8), 44.4% (*v*/*v*) glycerol, 4.4% SDS, and 0.02% bromophenol blue (Bio-Rad Laboratories, Inc., Hercules, CA, USA), and heated for 10 min in boiling water. A 12% separating gel and 4% stacking gel were prepared and used to separate proteins via gel electrophoresis. 5 μL of the molecular weight marker was deposited in the first well and 12 μL of the protein supernatants were deposited in the remaining wells. Electrophoresis was conducted at room temperature under the following conditions: 200 V, 25 mA, 250 W. After protein separation, the gel was stained with Coomassie Brilliant Blue R250 (Bio-Rad Laboratories) and subsequently destained overnight to allow for visualization of the protein distribution.

### 2.6. Rapid-Visco Analysis

The viscosity of the PPIs, PPC, and raw recipes was measured to characterize rheology behavior differences between pea proteins. Evaluation of samples was assessed according to AACC method 76-21 STD 1 [17], using the Rapid Visco Analyzer (RVA) 4800 (Perten Instruments, Perkin Elmer, NSW, Australia). Samples were hydrated by combining approximately 3.5 g of recipe with 25 mL of water, to obtain 14% (*w*/*v*), solids basis; dry sample amount was adjusted accordingly to account for the inherent moisture differences between recipes. Hydrated samples were placed in the RVA chamber and heated to 50 °C while being stirred under a constant shear rate of 960 rpm for 10 s. It was held at 50 °C for 50 s and then heated up to 95 °C under a shear rate of 160 rpm. It was held there for 3 min and then cooled back down to 50 °C. Peak and final viscosity values were captured for each sample. Pasting time and temperature were also evaluated. Testing was completed in triplicate.

### 2.7. Least Gelation Concentration

Gelling capability was characterized by least gelation concentration (LGC) for each PPI and PPC, using slight modifications of the method described by Zhu et al., (2017) [18]. This was conducted by dispersing different concentrations of each PPI and PPC (12–20% *w*/*v*) in DI water, heating at 95–100 °C for 1 h, immediately cooling in a cold-water bath, and transferring to a refrigerator at 4 °C for 2 h. LGC was determined, after chilling, as the minimum concentration of protein that forms a stable gel that does not fall or run upon inversion of the test tube.

### 2.8. Differential Scanning Calorimetry

Denaturation of the PPIs and PPC was determined using a Differential Scanning Calorimeter (DSC) Q100 (TA Instruments Inc., New Castle, DE, USA), according to the method of Brishti et al., (2017) [19], with slight modification. The instrument was calibrated using an empty pan as a reference. 5–10 mg of protein was weighed into a stainless-steel pan and hermetically sealed. The pan was heated from 25 °C to 250 °C at a rate of 10 °C/min. Each sample was measured in triplicate. Onset, peak denaturation, and end temperatures were recorded.

### 2.9. Size Exclusion Chromatography by High Performance Liquid Chromatography (SEC-HPLC)

The molecular weight distribution of pea proteins, raw recipes, and extruded recipes was estimated by size exclusion chromatography, using an Agilent 1100 HPLC system (Santa Clara, CA, USA), equipped with a Phenomenex SEC-4000 column (7.8 × 300 mm, Phenomenex, Torrance, CA, USA). To prepare the extruded recipes for analysis, the samples were dried for 48 h at −105 °C, 0.005 mbar, in a freeze dryer (FreeZone 4.5 Liter Benchtop Freeze Dry System, Labconco, Kansas City, MO, USA). Following, the dried samples were ground into a fine powder using a coffee mill (Casara coffee grinder, Model: SP-7440) for 30 s. To extract the protein, samples were dissolved at 1 mg/mL in 0.05 M sodium phosphate buffer (pH 6.8) containing 2% SDS (*w*/*v*). After shaking for 1 h at 250 rpm and centrifugation at 8000× *g* for 5 min, the supernatant was collected and filtered through a 0.45 μm Nylon membrane (Fisher Scientific, Hampton, NH, USA). Then, 20 μL of each sample was injected into the system for separation. The column temperature was maintained at 30 °C. The mobile phase, including 0.1% trifluoroacetic acid in water (phase A) and acetonitrile (phase B), was set at the following gradient conditions: 20–30% phase B at 0–20 min, 30–35% phase B at 20–25 min, and 35–20% phase B at 25–40 min to elute the residues. The proteins were separated at a flow rate of 0.7 mL/min and detected at 214 nm using a diode array detector (Agilent, Santa Clara, CA, USA). Protein standards, including thyroglobulin bovine (670 kDa), γ-globulins from bovine blood (150 kDa), and chicken egg grade VI albumin (44 kDa), were used as molecular weight references and analyzed at the same chromatography conditions.

### 2.10. Extrusion Processing

**Figure 1:**
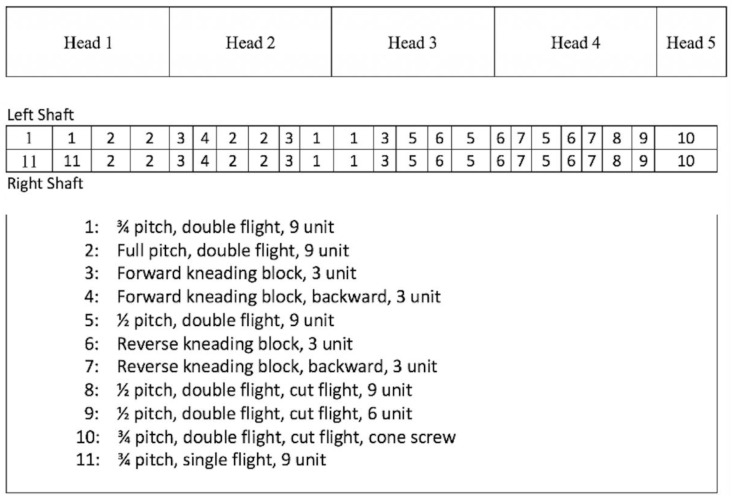
Extruder screw profile for extrusion of high-moisture meat analogs (HMMAs).

Prior to extrusion, each recipe was mixed for 5 min using a batch ribbon blender (Wenger Manufacturing, Sabetha, KS, USA). Processing was completed on a pilot-scale twin-screw extruder (TX-52, Wenger Manufacturing), equipped with a differential diameter cylinder preconditioner with a volumetric capacity of 0.056 m^3^ (DDC2, Wenger Manufacturing). Water was injected into the preconditioner at a rate ranging from 12–16 kg/h, and into the extruder at a rate ranging from 2–9 kg/h, depending on recipe requirements. The recipes were fed into the preconditioner at 35 kg/h, and then subsequently transferred into the extruder having a screw speed fixed at 450 rpm. The screw profile can be observed in Figure 1. Five heating zones were utilized to heat the extruder barrel; their respective temperatures were set at 25, 110, 110, 130, and 135 °C, moving from inlet to discharge end of the extruder. Other critical process parameters such as in-barrel moisture, specific mechanical energy, etc. are provided in Table 2. A long cooling slit die, with dimensions 48″ × 6″ × ½″ (L × W × H), was attached to the discharge end of the extruder to allow for protein alignment and texturization, and enable product cooling. The first section was run without cooling while the second die section was cooled with water, directly controlled by a manual throttle valve. Product was cut by hand at the die exit, into approximately 1 ft long pieces, with a Chef’s knife. All collected samples were immediately transferred to plastic totes and stored frozen until analysis.

A wattmeter, equipped to the extruder motor, was used to directly measure the mechanical energy required to turn the extruder screws. Specific mechanical energy (SME) was assessed from the power input given in kW. This was converted to kJ/kg with the following formula:

(2) $$\mathrm{SME}\;\left(\mathrm{kJ}/\mathrm{kg}\right)=\frac{\left(P-P_{0}\right)}{\frac{\begin{array}{c} \dot{m} \end{array}}{3600}}$$

where P is motor power reading in kW, P_0_ is the no load motor power reading in kW, and ṁ is mass flow rate in kg/h.

In-barrel moisture (IBM) and die cooling water injection rate were optimized for effective processing of each product. The IBM requirement stayed relatively constant between recipes (40–42% w.b.) but was slightly higher (44–45% w.b.) for the two recipes containing highest levels of PPI. This was adjusted to facilitate flow of the material through the extruder and to optimize texturization of each product.

IBM content was calculated using the following equation:

(3) $$\mathrm{IBM}\;\left(\%\;\mathrm{wb}\right)=\frac{\left(m_{f\;}\times {\;X}_{\mathrm{fw}}\right)+m_{\mathrm{pw}}+m_{\mathrm{ew}}}{m_{f}+m_{\mathrm{pw}}+m_{\mathrm{ew}}}$$

where m_f_ is the dry feed rate in kg/h, X_fw_ is the moisture content of the dry feed material, m_pw_ is the water injection rate into the pre-conditioner in kg/h, and m_ew_ is the water injection rate into the extruder in kg/h.

### 2.11. Texture Analysis

Cutting test and texture profile analysis (TPA) were utilized to evaluate the overall texture and physical properties of the extruded HMMA products. Evaluation of samples was performed using a TA-XT2 Texture Analyzer (Texture Technologies Corp., Scarsdale, NY, USA). Prior to analysis, the frozen, extruded products were thawed in a 2% saltwater brine at room temperature for 20 min to prevent desiccation. Former research by Kim et al., (2021) [20] supports this method for maintaining consistency and quality in product texture during thawing.

The cutting strength was measured in both the longitudinal and transverse directions, similar to the procedures described by Zahari et al., (2020) [21]. Longitudinal is parallel to the direction of material flow through the extruder die while transverse is perpendicular to direction of die flow. Samples were cut into squares with dimensions of 3 × 3 cm and then analyzed. A guillotine knife blade (70 mm width × 100 mm height × 3 mm thickness), at a test speed of 3 mm/s, was used to cut through the entire height of the sample, from top to bottom, in all cases. The maximum force required and the work necessary to achieve this (i.e., the area under the curve) were taken as an index of firmness and toughness, respectively, of the sample. Four animal meat anchors (beef steak, pork chop, chicken breast, salmon fillet) were also measured for texture comparison, using the same test procedures. The meat anchors were thawed in a refrigerator overnight, then cooked on a Proctor Silex nonstick electric griddle at 177.7 °C (350 °F) to internal temperatures recommended “as safe” by USDA [22]. Products were flipped every 10 min until done. HMMA samples were thawed, but not heated like the animal meat anchors, prior to texture analysis. For the animal meat products, the longitudinal cut was considered as the direction parallel to the elongated muscle fibers while the transverse cut across the myofibril fibers.

10 replicates were measured for all extruded products and meat anchors for each direction of cut. Data was collected for firmness and toughness values.

TPA was also conducted on both the extruded HMMA products and animal meat anchors. Samples were cut into squares with dimensions of 3 × 3 cm, as described above for cutting test. A 50 mm diameter aluminum cylinder probe compressed the sample twice to 25% strain, with a contact force of 1000 g, at a test speed of 2 mm/s, and a wait time of 2 s between each compression. Meat anchors, cooked following same procedures done for cutting test, were also measured using TPA, for comparison. Fifteen replicates were measured for each sample. Data was collected for attributes of hardness, resilience, cohesiveness, and gumminess for all products [23].

### 2.12. Sensory Analysis

A descriptive sensory analysis was performed on HMMA extrudates and animal meat anchors. Six samples were evaluated in total. PPI3-40, PPI4-40, PPI1-30, and PPI1-60 were the HMMA products selected for analysis. In addition, beef steak (beef loin, choice, KC strip steak, boneless) and chicken breast (chicken breast, skinless, boneless, 99% fat-free) were purchased from Dillon’s Food Store (Manhattan, KS, USA), for sensory evaluation as well.

Prior to analysis, HMMAs and animal meat anchors were heated and cooked, respectively, using a non-stick skillet on a gas stovetop. HMMA samples were thawed in room-temperature 2% saltwater brine for 30 min, submerged in vegetable oil, and then transferred to a heated skillet. Products were heated for 4 min at 177.7 °C (350 °F), with samples being flipped once after 2 min. Animal meat anchors were thawed overnight in a refrigerator, and then transferred to a skillet and cooked to safe temperatures as recommended by USDA [22]. After cooking, samples were transferred to microwaveable bowls and sealed with aluminum foil to keep warm. Prior to serving, all samples were cut into ½” cubes and were warmed in a microwave on high power for 30 s. Individual analysis of the samples was done in duplicate using 4 highly trained professional sensory panelists. All panel members were female. In regard to the number of panelists, the Society of Sensory Professionals (SSP), suggests that the appropriate number of panelists can vary depending on the study and the level of training. It is mentioned that 4–18 panelists have been previously reported, but that this should be justified based on their training and experience [24,25]. The individuals had an initial 120 h of sensory descriptive analysis panel training for a variety of food products. Subsequently they were involved in sensory studies on an ongoing basis for various product categories, allowing them to gain extensive experience in descriptive analysis having conducted more than 1000 h of sensory testing/evaluation on a variety of food products, including animal meat, as well as other animal and vegetable products. The study was reviewed and approved by the Kansas State University Institutional Review Board. The panelists went through a 2 h. orientation session one day prior to analysis to allow them to finalize the lexicon, familiarize themselves with the products, and understand the characteristics of HMMA products. Eight pieces of each sample were served to each panelist in 3.25 oz containers, each being blindly labeled with a random 3-digit code. The panelists tasted the product and then rated each attribute intensity using a 15-point scale with 0.5 increments, where 0 meant “none” and 15 meant “extreme”. A total of 17 attributes were identified, defined, and referenced. These attributes are described in detail in the Section 3. Panelists were provided unsalted crackers and deionized water for palate cleansing between samples.

### 2.13. Statistical Analysis

Data from analysis was evaluated using IBM SPSS Statistics version 25.0 software (SPSS Inc., Chicago, IL, USA), XLSTAT (Addinsoft Incorporated, New York, NY, USA), and SAS software (SAS, Cary, NC, USA). All means and pair-wise differences were calculated using 1-way ANOVA and Tukey’s test. Results with significance level of (*p* < 0.05) were considered to be statistically significant. Principal Component Analysis (PCA) was also conducted for latent pattern discovery through data reduction and dimension exploration.

## 3. Results and Discussion

### 3.1. Protein Content

As shown in Table 2, PPI1 had the highest protein content (80.00%), followed by PPI3 (78.75%), PPI2 (77.19%), and PPI4 (76.88%). The protein content of PPC was significantly lower than the PPIs (53.22%).

### 3.2. Particle Size

Particle size is reported to play a significant role in low-moisture extrusion, especially in regard to the processing and texture-forming properties of the ingredients [9]. Hence, the particle size was measured for each of the PPIs and PPC. Their cumulative distributions are shown in Table 3. Of the different PPIs, PPI1 had the largest particle size, followed closely by PPI4. At 53–63 μm, differences in particle size between PPIs and PPC became apparent. Greater than 90% of the particles for PPI2, PPI3, and PPC were smaller than 63 μm, while only 30% of the particles were smaller for PPI1 and PPI4. The variation in particle size is due to differences in pea protein grinding methods [9].

### 3.3. Protein Solubility

**Figure 2:**
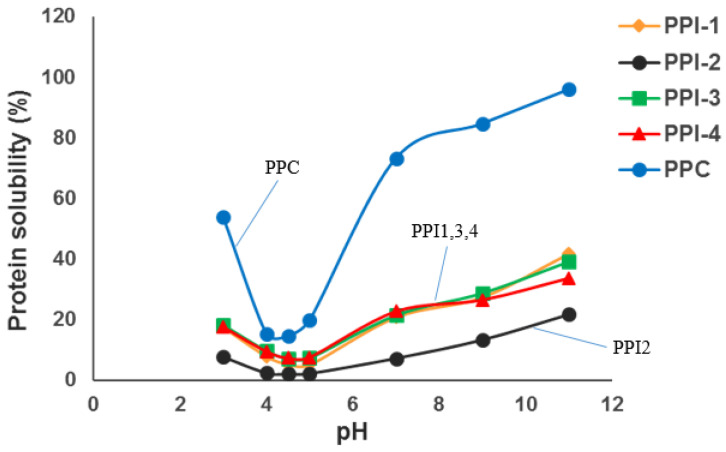
Protein solubility curves of pea protein isolates (PPIs) and pea protein concentrate (PPC).

As expected [26], minimal solubility was observed for all pea proteins at the isoelectric point around pH 4.5, with solubility increasing when the pH was further increased or decreased beyond the isoelectric point (Figure 2). PPC had the highest solubility at each pH value while PPI2 had the lowest solubility. PPI1, PPI3, and PPI4 possessed similar protein solubility trends from pH 3 to 11. According to Osen et al., (2014) [9], protein solubility reflects the heat treatment history of proteins, with a lower solubility following extensive heat treatment from mechanical grinding or high-temperature spray-drying.

### 3.4. Sodium Dodecyl Sulphate-Polyacrylamide Gel Electrophoresis

**Figure 3:**
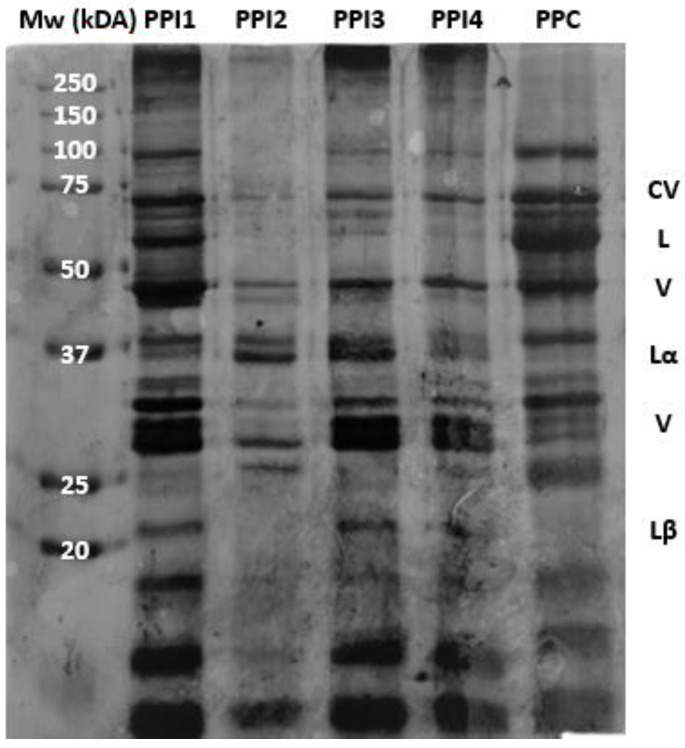
Bands of pea protein isolates (PPIs) and pea protein concentrate (PPC) from sodium dodecyl sulphate–polyacrylamide gel electrophoresis (SDS-PAGE), under reducing conditions. Mw = molecular weight markers. Bands of CV = convicilin, L = legumin, V = vicilin, Lα = Legumin alpha, and Lβ = Legumin beta are identified across lanes.

The molecular weight profiles of the PPIs and PPC were analyzed via SDS–PAGE. Results are shown in Figure 3. Pea proteins are comprised of two major components, legumin and vicilin, as well as a small amount of convicilin [27]. All bands show the presence of each of these components, although variation in color intensity is apparent. The intensity of the lines suggests differences in protein solubility. Darker bands indicate higher solubility while lighter bands indicate lower solubility [27,28,29].

**Figure 4:**
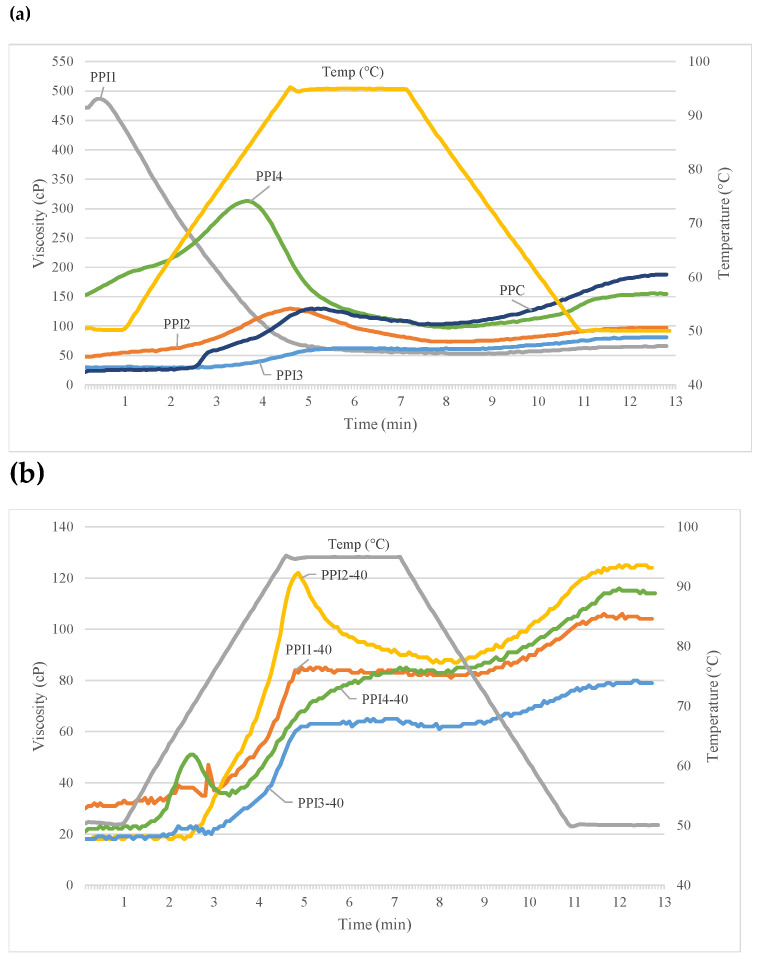
Rapid-visco analysis curves for (**a**) pea protein isolate (PPI) types and pea protein concentrate (PPC) and (**b**) high-moisture meat analog (HMMA) recipes containing different pea protein isolate (PPI) types.

PPI3 and PPI4 contain almost identical bands, providing inference for their similar processing conditions during extrusion. PPI2 profile is nearly transparent, with darker bands only appearing at Lα and V. The combination of fewer and lighter bands suggests lower protein solubility for PPI2 [28,29], while the darker bands for PPC suggests higher protein solubility; this was confirmed using the Biuret method (see Figure 2). Darker bands are also noted in PPI1, indicating higher solubility; the cold-paste solubility identified using RVA (Figure 4a) provides confirmation for its high solubility. Overall, the differences in SDS-PAGE profiles between the pea proteins are related to the plant species, protein extraction methods, and previous processing history [27].

### 3.5. Rapid-Visco Analysis

The Rapid-Visco Analyzer (RVA) was used to measure rheology differences among the individual pea proteins and raw recipes (Figure 4 and Table 4). Distinctions in flow properties were evident between the various PPIs when subjected to heat, moisture, and shear. The pasting profiles relate to the proteins’ functional properties, namely protein solubility, water binding capacity, and heat gelation [9]. Figure 4a is a collective graph including one replicate of each PPI and PPC run on the RVA.

As shown in Table 4, PPI1 showed high initial cold-water viscosity, with an average peak reaching 525 cP, that immediately decreased upon heating to 95 °C; however, average final viscosity for PPI1 was lowest among all PPIs, at 68 cP. Upon hydration, this protein powder absorbs water and swells, resulting in a high starting viscosity that subsequently decreases with the addition of thermal energy and shear [9]. It was also observed that PPI1 also led to the highest SME among PPI types (Table 2). PPI4 demonstrated hot-paste viscosity, with an average peak occurring at 293 cP after heating, which subsequently declined to a final viscosity of 145 cP. In contrast, PPI2 and PPI3 had low starting viscosities, that marginally increased during heating to 95 °C. The low starting viscosity can be attributed to lower protein solubility and particle size [9], relative to PPI1 and PPI4. High solubility of PPI1 and low solubility of PPI2 is the same as found in SDS-PAGE. These observations demonstrate functionality differences between the PPI types.

Initial viscosity is likely attributed to protein solubility, while sudden increases in viscosity after heating, e.g., PPI4, relate to heat gelation properties. The superior heat-gelling ability of PPI4 is confirmed by LGC. It should be noted that higher protein solubility is desired for better functionality and texturization in low-moisture extrusion of TVP [8,30]; however, this is not always true for high-moisture extrusion applications, like HMMA. In general, the PPIs with lower solubility led to HMMAs with better visual quality and uniform texture.

**Figure 5:**
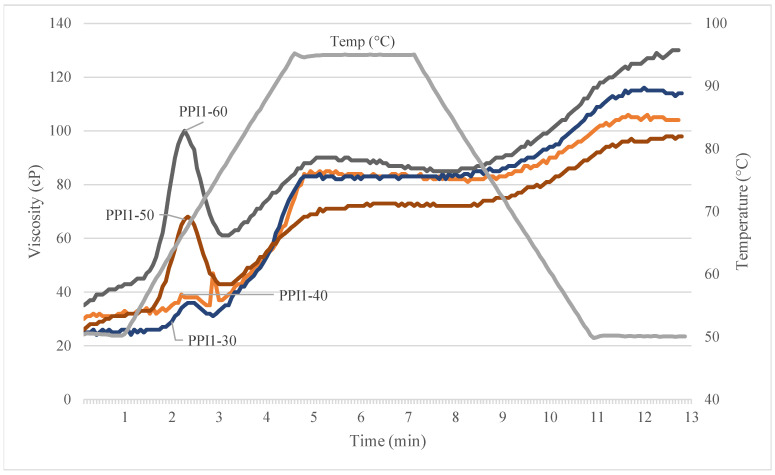
Rapid-visco analysis curves of high-moisture meat analog (HMMA) recipes containing different inclusion levels of pea protein isolate (PPI) and pea protein concentrate (PPC). Peak viscosity of PPI1-60 was significantly higher than PPI1-30, PPI1-40, and PPI1-50. No significant difference in peak viscosity was observed between PPI1-30 and PPI1-40.

Recipes show quite different RVA curves due to the addition of concentrate, flour, and fiber (Figure 4b and Figure 5, Table 4). In general, the addition of these nutrients lowered the solubility and viscosity of the curves, compared to the individual pea proteins. All curves began at a low initial viscosity (~20–30 cP), but upon heat and shear addition, viscosity increased with time. PPI3-40 showed lowest peak viscosity (23 cP), which was consistent with Figure 4a. The heat gelation property of PPI4 is again identified by a small peak occurring at ~150 s, after heating.

Peak viscosity increased with higher PPI inclusion (32-91 cP), as found in Figure 5; this observation was the same as found in Onwulata et al., (2014) [31]. Molecular weight of the recipes increases with greater substitution of PPI for PPC, inducing higher viscosity. As concentrate is substituted for isolate, overall molecular weight decreases, and peak viscosity is less. These results pair well with specific mechanical energy (SME) values. Lower viscosity is generated with lower PPI inclusion (30–40%); therefore, higher SME (946–1080.00 kJ/kg), by way of lower water input (40.57–41.99%), is required to achieve optimum product quality. The recipes higher in PPI inclusion (50–60%) generate higher viscosity, so less SME (761–781 kJ/kg) is needed to induce texturization and obtain optimum product quality.

### 3.6. Least Gelation Concentration

LGC test was performed on the PPIs and PPC. Results are displayed in Table 5. (+) symbol indicates a weak gel, while (++) symbol indicates a strong gel. Alternately, (-) symbol signifies no gelling occurred, while (/) symbol denotes an increase in viscosity but no firm gelling. Proteins exhibiting lower LGC are said to have greater heat-gelling capacity [32]. Based upon the results, PPI1 did not increase viscosity or form a gel until reaching 16% protein concentration (*w*/*v*); for this reason, LGC was considered highest for PPI1. PPI2 and PPI3 increased viscosity at 14% (*w*/*v*) but did not fully form rigid gels until 16%. PPI4 and PPC exhibited lowest LGC at 12% protein concentration (*w*/*v*).

The low LGC for PPC and PPI4 was primarily caused by their higher heat-gelling capacities [29], which was consistent with RVA. The greater heat gelation capacity of PPI4, relative to the other PPIs, corresponds to its hot peak viscosity in RVA. PPI1 exhibited highest cold peak viscosity, but viscosity decreased sharply upon addition of heat, providing indication of lower heat gelling capacity. Nicolai & Chassenieux (2019) [33] discovered that higher cold-water solubility can be achieved through protein hydrolysis but this results in a tradeoff of lower gel strength. Thus, PPI1 may have been processed in a way to increase its cold-water solubility but was achieved at the expense of gel strength; this was confirmed by highest LGC found in PPI1. These results demonstrate that LGC, in combination with RVA, can be used to characterize proteins into cold swelling and heat swelling properties [29]. In a recent publication by Tulbek et al., (2017) [34] (pp. 145–164), it is noted that proteins having a lower gelation concentration possess a greater ability to bind water and fat. This could interrupt protein crosslinking and may have led to the weaker internal texture as seen in PPI4-40 and PPI1-30.

For future studies, the ‘bloom strength’ of gels could be measured to quantitatively evaluate gel strength. Liu et al., (2019) [35] (pp. 441–463) gives detailed insight into specific methodology and instrumentation that could be used to perform this.

### 3.7. Differential Scanning Calorimetry

DSC was used to measure the denaturation temperature of the pea proteins. It is important to know this value prior to extrusion, as protein cross-linking will only occur above this temperature [19]. Denaturation is identified by a change in heat enthalpy, which takes on the form of a peak. Results, shown in Table 6, were different among PPI types. PPI1 showed significantly highest denaturation and end temperatures (190.82 °C and 206.75 °C, respectively), while PPC showed significantly lowest denaturation temperature (175.79 °C). The denaturation temperatures are linked to the heat-gelling properties of the proteins. Denaturation temperature was highest for PPI1 and it possessed the poorest heat swelling properties as shown by RVA and LGC. In contrast, PPI4 and PPC have greatest heat swelling capability but lowest denaturation temperatures. The proteins with better heat-swelling capabilities are able to reach peak denaturation temperature much quicker by way of protein aggregation and more efficient heat transfer.

### 3.8. Size Exclusion Chromatography by High Performance Liquid Chromatography (SEC-HPLC)

**Figure 6:**
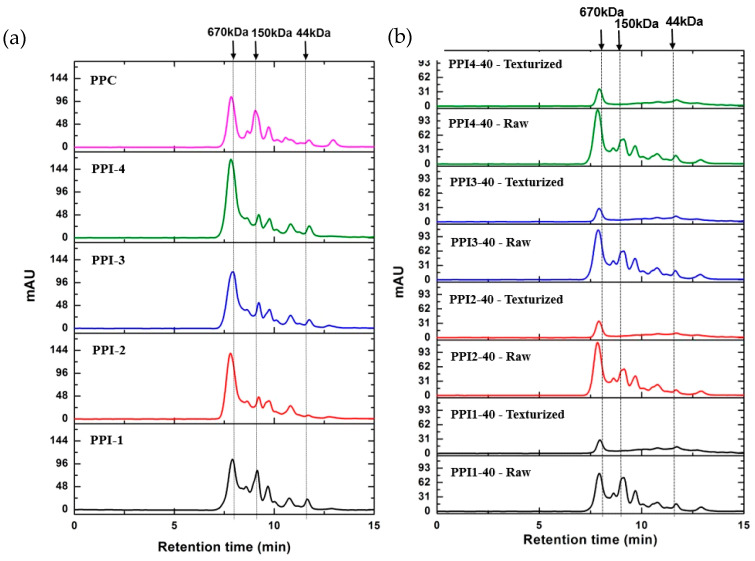
Size exclusion high performance liquid chromatography (SEC-HPLC) separation of (**a**) pea protein isolate (PPI) types and pea protein concentrate (PPC) and (**b**) raw recipes, differing in PPI type, and their respective high-moisture meat analog (HMMA) extrudates into peptide fragments.

PPC had a higher proportion of lower molecular size proteins, as indicated by the relatively smaller absorbance peak around 670 kDa and larger peak at 150 kDa (Figure 6a). The profile of PPC was comparable with PPI1. PPI2, PPI3, and PPI4 shared similar peak patterns, being composed of larger molecular sized proteins, with a dominant peak around 670 kDa. A correlation between protein size and heat-gelling capacity was observed, specifically for PPI1 and PPI4. LGC was observed to be highest for PPI1 and lowest for PPI4; furthermore, PPI1 was more fractionated, i.e., contained more small-sized proteins, while less fractionation was observed in PPI4. Webb (2021) [29] cited that stronger gels are often seen in pea proteins that are less fractionated. The high degree of fractionation found in PPI1, using SEC-HPLC, is a potential indicator for its poor heat-gelling capability.

SEC-HPLC results from the raw recipes, and their respective texturized extrudates, are presented in Figure 6b. PPI1-40 raw had a relatively higher proportion of lower molecular size proteins. This is evidenced by a relatively smaller absorbance peak around 670 kDa and a larger peak around 150 kDa, relative to PPI2-40, PPI3-40, and PPI4-40. This is consistent with results from Figure 6a. After texturization, protein solubility decreased for all proteins dramatically, with much smaller peaks presented at 670 kDa, and disappearance of the majority of the smaller proteins found in the raw recipes. The relatively flat chromatograms, after 670 kDa, in the texturized samples, indicate lower solubility of the proteins, which is created by texturization, via cross-linking [29].

SEC-HPLC results for raw recipes and texturized products for different levels of PPI1 inclusion are not shown, but the primary observation from those results was that with an increase of PPI1 inclusion, particularly at 50 and 60%, a small peak at 44 kDa appeared after extrusion. This would imply incomplete texturization and/or protein cross-linking with higher amount of PPI in the formulation. The upper separation limit of the Phenomenex SEC-4000 column is around 700 kDa, which may explain why the newly cross-linked proteins in the texturized products were not identified through the SEC-HPLC.

### 3.9. Extrusion Parameters

#### 3.9.1. Moisture

IBM was optimized between recipes to target high-quality HMMA products, based on visual evaluation. As shown in Table 2a, no major adjustments in IBM content (%) among the four PPI types were required. Prior research supports evidence that there is a positive correlation between IBM and protein inclusion [36]. At the lowest protein inclusion, i.e., PPI1-30, less IBM was required (40.57%). As PPI inclusion level increased, as seen in Table 2, IBM was increased as well (40.57–45.31%). Higher protein, by way of substituting PPI for PPC, requires more water for processing, given the increase in molecular weight [6].

#### 3.9.2. Mechanical Energy

System response variables, such as die pressure, die temperature, and SME, are indicators of melt viscosity and are usually related to the quality of the final extrudate [37,38]. These values result from the combined interaction of independent process parameters and recipes. Given that the extrusion parameters were kept constant, apart from IBM, changes in mechanical energy are primarily driven by a three-way interaction between IBM, protein inclusion amount, and functional differences among the PPI types.

SME is a measurement that allows mechanical energy to be quantified. These values, shown in Table 2, correlate well with IBM and PPI inclusion. Lowest SME, die pressure, and die temperature were shown for PPI-50 and PPI-60, which were (a) processed at highest IBM and (b) contained highest overall protein inclusion. PPI1-30 contained lowest overall protein inclusion (51.33%) and was processed at the lowest IBM (40.57%). More mechanical energy was needed to achieve optimum product quality with lower inclusion of PPI. As the PPI inclusion level increased, the level of SME needed to reach optimum product quality was lower given the positive effect of PPI on texturization.

SME ranged from 699.43 to 812.57 kJ/kg amongst PPI1-40, PPI2-40, PPI3-40, and PPI4-40. These slight differences are likely related to functional differences in protein solubility, denaturation temperature, heat-gelation properties, and protein content of the PPIs. SME was highest for PPI1-40, while die temperature and pressure were lowest for PPI4-40. PPI1 exhibited highest cold-paste solubility while PPI4 possessed greatest heat-gelling capacity.

### 3.10. Texture Analysis

#### 3.10.1. Cutting Test

**Figure 7:**
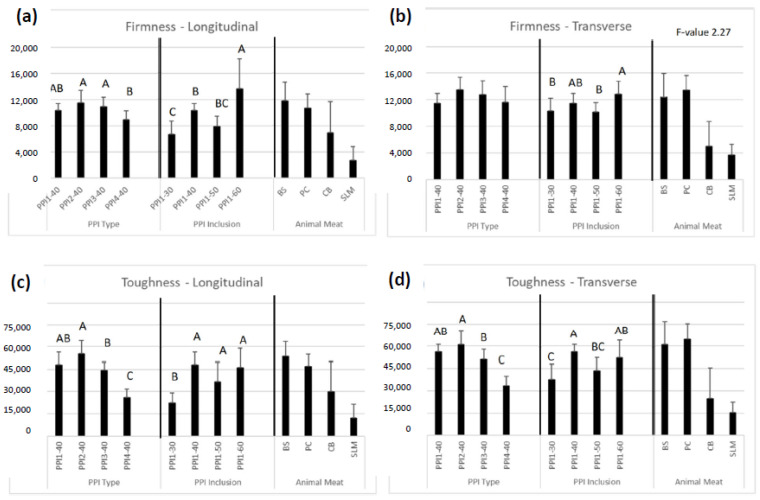
Cutting test results of high-moisture meat analog (HMMA) products differing in pea protein isolate (PPI) type and PPI inclusion, and also animal meat anchors for (**a**) longitudinal firmness, (**b**) transverse firmness, (**c**) longitudinal toughness, and (**d**) transverse toughness. Within each individual bar cluster, bars labeled with the same letter are not significant.

Cutting test results are shown in Figure 7. Among recipes differing in PPI type, PPI2-40 displayed highest firmness (11,530–13,550 g) and toughness values (55,190–60,965 g. s), having significantly higher toughness than PPI3-40 (44,620–50,970 g. s) and PPI4-40 (26,110–33,725 g. s). PPI4-40 exhibited lowest firmness and toughness compared to all other HMMAs differing in PPI type.

**Figure 8:**
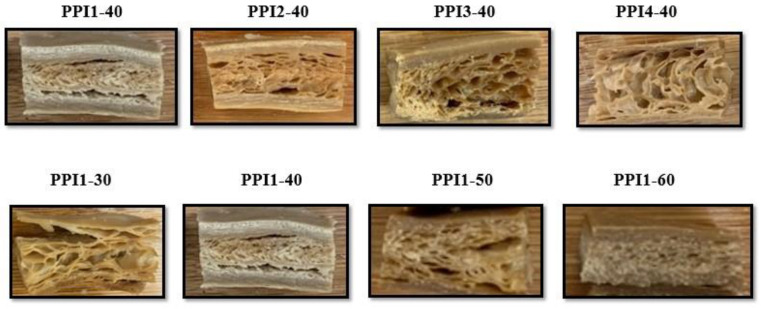
Cross-section photographs of extruded pea-based high-moisture meat analog (HMMA) products.

Almost all recipes, apart from PPI4-40 and PPI1-30, displayed similar firmness and toughness values to the beef steak and pork chop. Given their soft texture, PPI4-40 and PPI1-30 were more similar to the chicken breast, especially in regards to toughness. The HMMAs were too firm and tough to match the softness of salmon. Transverse to longitudinal cutting ratios are observed in Table 7. All ratios are greater than 1, except for PPI1-60, emphasizing a high degree of isotropic, intramolecular bonding, compared to anisotropic, intermolecular bonding. A greater amount of intramolecular bonding, i.e., protein crosslinking occurring between sulfur amino acids on the same polypeptide chain [39] (pp. 435–489), was achieved by complementing PPI with PPC. A product image of each HMMA can be seen in Figure 8; the outer shell created for pea-based recipes in prior experiments is shown to be greatly reduced after supplementing PPI with PPC.

In general, increasing the PPI inclusion level led to significantly higher product firmness, as in Webb et al., (2020) [6]; toughness differences were not as pronounced. With more protein present in the recipe, a higher degree of texturization occurs during extrusion.

#### 3.10.2. Texture Profile Analysis

**Figure 9:**
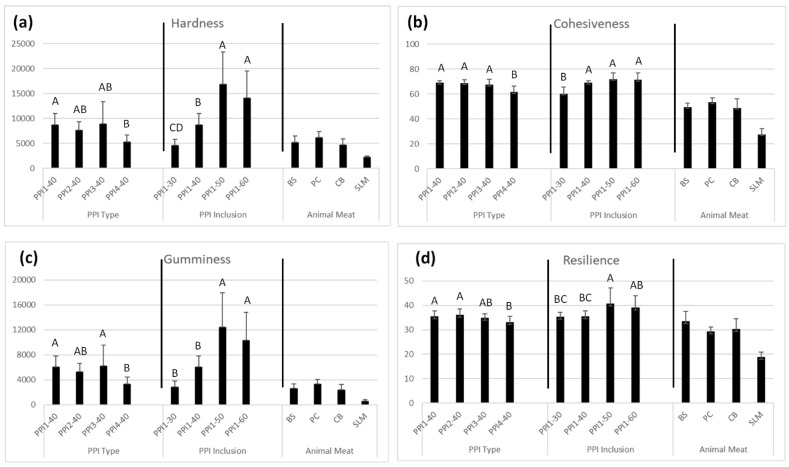
Texture profile analysis (TPA) results of high-moisture meat analog (HMMA) products differing in pea protein isolate (PPI) type and PPI inclusion, and also animal meat anchors for (**a**) hardness, (**b**) resilience, (**c**) cohesiveness, and (**d**) gumminess. Within each individual bar cluster, bars labeled with the same letter are not significant.

TPA results for hardness, resilience, cohesiveness, and gumminess are displayed in Figure 9. A high contact force (1000 g) was used to assess ‘true’ internal product structure and eliminate irregularities caused by differences in piece thickness and shape. Among the HMMAs using different PPI types, PPI1-40, PPI2-40, and PPI3-40 demonstrated higher hardness, resilience, cohesiveness, and gumminess values than PPI4-40. PPI-2 showed highest resilience (36%) and cohesiveness (69%) compared to PPI1, PPI3, and PPI4, which accords with the high cutting test values. PPI2 was lowest in protein solubility and led to highest cutting test and TPA values. PPI4 had greatest heat gelling capacity among PPI types and led to lowest texture values. These properties may be important to keep in mind for future HMMA studies.

In general, higher PPI inclusion also led to higher TPA values. PPI1-50 and PPI1-60 showed higher hardness, resilience, cohesiveness, and gumminess values compared to PPI1-30 and PPI1-40. All HMMA products showed statistically similar and/or higher values, compared to the animal meat anchors. PPI1-50 and PPI1-60 values were especially higher, confirming the significant effect that PPI inclusion has on product hardness and overall texturization. Unlike the animal meat anchors, the HMMA products did not undergo heating prior to analyzing texture. Therefore, many of the values were significantly higher compared to their animal meat counterparts. With additional culinary preparation, e.g., heating, most of the HMMA products could likely be softened to provide a better texture match to their animal meat counterparts. As the culinary preparation facet was not a primary focus of this study, future work in this area is needed to provide more definitive results. From these results, PPI4-40 and PPI1-30 are more suited to target a softer meat product like chicken breast, whereas the other recipes could be specialized to match a beef steak or pork chop, given their similar textural attributes. PPI1-50 and PPI1-60 are perhaps too hard to mimic animal meat.

HMMA structure and texture data indicated that protein functionality such as cold water solubility and heat gelation dictate texturization and final product quality. For example, PPI1 was unique among all ingredients as it has the highest cold water solubility and also LGC as was described in Section 3.5 and Section 3.6, respectively. This might have led to excessive protein cross linking and thicker yet less laminated shell or surface layer. Previous studies have demonstrated similar links between cold swelling and heat gelation properties of various plant proteins including those derived from yellow peas, soybeans and wheat (gluten), on one hand, and their cross-linking potential and texturization via extrusion on the other [6,40]. Although these studies were related to lower moisture based texturization (less than 35% wet basis in-barrel moisture), the same chemistry and thermodynamics-based food structure engineering principles apply to high-moisture meat analog processing. In the case of HMMA, the more porous and less laminated ‘shell’ structure of products based on higher cold swelling protein might have led to lower cutting firmness and toughness, thus less than desirable product texture as compared to animal meat benchmarks as discussed in this section. Conversely, PPI2 with less cold water solubility and lower LGC had more laminated surface layer, and higher cutting test and texture profile analysis response.

### 3.11. Sensory Analysis

Textural attributes evaluated were springiness, denseness, juiciness, residual particles, firmness, chewiness, fiber awareness, tooth packing, astringent, and starchy. The flavor attributes evaluated were beany, starchy, grain, green, umami, barnyard, and cardboard. The definitions and references used for each attribute are presented in Table 8. For example, springiness was defined as “the degree to which sample returns to its original height when compressed once partially with molar teeth and slowly released” (reference was an Oscar Mayer wiener given a score of 3.5). Firmness was “the force required to bite completely through the meat pieces with the molar teeth” while chewiness is “the difficulty with which the sample can be broken down with the molars for swallowing”. Both attributes used Hormel cured ham with a score of 8.5 and 7.5, respectively as a reference. In terms of flavor attributes, beany was defined as “a slightly brown, musty, slightly nutty and starchy flavor associated with cooked beans” (Bush’s pinto beans scored at 7.5 for reference). Umami was “a general term for aromatics associated with juices from cooked seafood, meat, and/or vegetables” and button mushroom broth given a score of 2.0 was used as its reference.

**Figure 10:**
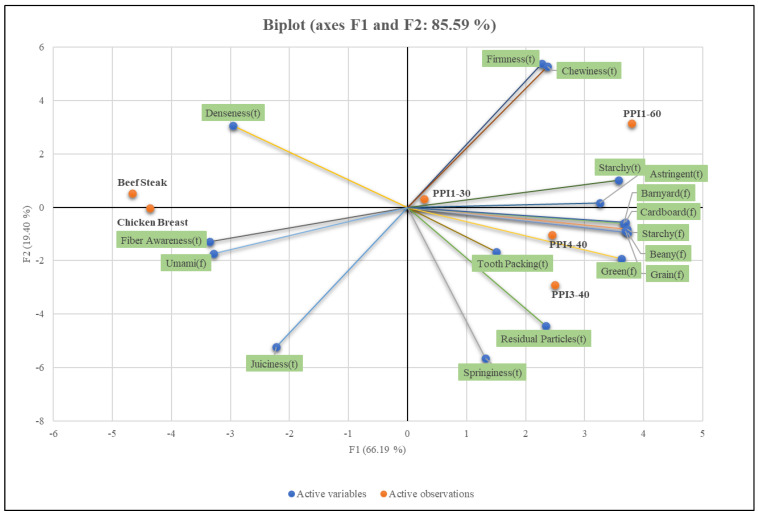
Principal component analysis (PCA) plot of least square (LS) means for texture and flavor attributes of pea-based high-moisture meat analogs (HMMAs) and animal meat anchors.

The trained panelists’ perceptions are detailed in Table 9 and Table 10. For texture attributes, no significant differences (*p* < 0.05) were noted for springiness, juiciness, residual particles, fiber awareness, tooth packing, or astringent properties among all products evaluated. Among the HMMA samples, PPI1-60 was reported to have the greatest chewiness (11.1) and firmness (13.0), while PPI3-40 was highest in springiness (5.2) and juiciness (5.9). The high firmness of PPI1-60 correlates well with texture analysis data, where cutting test showed firmness to be greatest for PPI-60 compared to the other HMMA samples. Relative to animal meat (beef steak and chicken breast), the PCA plot (Figure 10) shows PPI-30 being the most similar in texture to animal meat, followed closely behind by PPI3-40 and PPI4-40. The high springiness (5.2) of PPI1-30 led to the highest juiciness value (5.9) among HMMAs, that was reported to be most similar to beef steak (6.2) and chicken breast (5.6).

Differences in PPI inclusion were not reported to affect flavor attributes of the HMMAs nearly as significantly as texture. All HMMA samples were perceived to be significantly more beany than animal meat, while only PPI1-30 was statistically similar to animal meat with regards to starchy, grain, green, and cardboard flavor attributes. Pulse proteins have been known to increase the beany flavor of plant-based meat and various methods employed by industry for removal of these undesirable flavors from legumes have been documented [20,41]. As expected, the value of beany flavor increased from 3.7 to 4.9 as the inclusion amount of PPI increased from 30 to 60%.

Based on these results, it was perceived that the HMMA recipes containing higher PPI inclusion (60%) led to extruded products that were too firm to appropriately mimic the animal meat anchors. A recommendation for future studies is to select recipes with lower PPI inclusion (30–40%) and extrude them through a longer and thinner cooling die, to provide enough time for sufficient product cooling to occur. Figure 8 shows the formation of an outer shell present in some of the HMMA products, which is caused by inadequate cooling in the die.

## 4. Conclusions

The texture and quality of HMMAs depend heavily on the type of commercial PPI utilized, as well as its level of inclusion. Functional differences found between the PPIs are likely to be most attributed to the specific plant species and respective protein extraction method. These variables create differences in particle size, protein solubility, and molecular weight of the protein polymers, which changes their functionality. Protein solubility and heat gelation properties seemed to be the biggest differences observed among the pea proteins. Higher visual product quality and uniform texture was found to be associated to the PPIs with lower protein solubility (PPI2 and PPI3). With regards to overall protein content, a higher inclusion of PPI (50–60%) in the recipe was found to significantly increase product hardness, firmness, and overall texturization, likely created by an increased amount of protein crosslinking. PPI4, which had highest heat-gelling capacity, induced lowest texturization. Conversely, PPI2, which exhibited lowest protein solubility, led to the greatest amount of texturization. In many cases, the HMMA textural attributes were similar to beef steak, pork chops, or chicken breast, but the salmon fillet was too soft to mimic. For future studies, systematic changes to extrusion process parameters and die design should be assessed to understand their collective impact on the final product texture of HMMAs.

## Figures and Tables

**Table 1:** Pea-based high-moisture meat analog (HMMA) recipes containing (a) different pea protein isolate (PPI) types and (b) different inclusion levels of PPI and pea protein concentrate (PPC).

	Recipes a				Recipes b			
Ingredients:	PPI1-40	PPI2-40	PPI3-40	PPI4-40	PPI1-30	PPI1-40	PPI1-50	PPI1-60
Pea Protein Isolate	40	40	40	40	30	40	50	60
Pea Protein Concentrate	38	38	38	38	48	38	28	18
Pea Flour	13	13	13	13	13	13	13	13
Pea Fiber	5	5	5	5	5	5	5	5
Salt	2	2	2	2	2	2	2	2
Oil	2	2	2	2	2	2	2	2
Total	100	100	100	100	100	100	100	100

**Table 2:** Total estimated nutritional content and extrusion processing conditions for each pea-based high-moisture meat analog (HMMA) recipe, differing in (a) pea protein isolate (PPI) type and (b) inclusion level of PPI and pea protein concentrate (PPC). Protein content was measured for each respective PPI: PPI1 (80.00%), PPI2 (77.19%), PPI3 (78.75%), PPI4 (76.88%), and PPC (53.22%), and overall nutritional content was estimated accordingly.

	Recipes a				Recipes b			
Nutrients:	PPI1-40	PPI2-40	PPI3-40	PPI4-40	PPI1-30	PPI1-40	PPI1-50	PPI1-60
Protein (%)	54.00	52.88	53.50	52.76	51.33	54.00	56.68	59.36
Starch (%)	10.36	10.36	10.36	10.36	10.56	10.36	10.16	9.96
Fiber (%)	8.62	8.62	8.62	8.62	10.12	8.62	7.12	5.62
Fat (%)	1.65	1.65	1.65	1.65	2.05	1.65	1.25	0.85
**Extrusion Conditions:**								
In-Barrel Moisture (%)	41.99	42.52	41.01	41.12	40.57	41.99	44.64	45.31
Specific Mechanical Energy (kJ/kg)	812.57	750.86	699.43	709.71	678.86	812.57	514.29	493.71
Die Temperature (°C)	163	161	163	158	161	163	155	151
Die Pressure (psig)	850	825	650	575	575	850	400	425

**Table 3:** Particle size distribution for pea protein isolate (PPI) types and pea protein concentrate (PPC).

	Cumulative (%)				
Sieves (μm)	PPI1	PPI2	PPI3	PPI4	PPC
25	1.5	3.9	4.2	2.3	0.3
32	5.2	11.5	4.9	4.8	1.4
53	27.8	81.5	93.4	23.3	71.6
63	33.8	92.1	95.1	33.4	92.3
75	41.4	96.4	96.2	43.2	99.9
90	50.7	98.6	97.1	54.6	100.0
106	57.9	99.5	97.7	63.0	100.0
125	66.0	100.0	98.2	71.4	100.0
250	96.2	100.0	99.9	97.3	100.0

**Table 4:** Rapid-visco analysis of pea protein isolate (PPI) types, pea protein concentrate (PPC) and pea-based high-moisture meat analog (HMMA) recipes containing different pea protein isolate (PPI) types. Cells labeled within each column with the same letter are not significant.

	Peak Viscosity (cP)	Final Viscosity (cP)	Pasting Time (s)	Pasting Temp (°C)
PPI1	525 ± 59 ^A^	68 ± 3 ^D^	24 ± 11 ^E^	50 ± 0 ^C^
PPI2	115 ± 14 ^C^	90 ± 6 ^C^	293 ± 5 ^C^	95 ± 0 ^A^
PPI3	63 ± 2 ^C^	83 ± 3 ^CD^	349 ± 0 ^A^	95 ± 0 ^A^
PPI4	293 ± 19 ^B^	145 ± 9 ^B^	232 ± 0 ^D^	85 ± 0 ^B^
PPC	133 ± 5 ^C^	182 ± 5 ^A^	312 ± 0 ^B^	95 ± 0 ^A^
**Recipe**				
PPI1-40	39 ± 7 ^BC^	106 ± 5 ^A^	153 ± 6 ^B^	69 ± 1 ^B^
PPI2-40	118 ± 9 ^A^	117 ± 8 ^A^	304 ± 4 ^A^	95 ± 0 ^A^
PPI3-40	23 ± 1 ^C^	81 ± 10 ^B^	165 ± 16 ^B^	71 ± 3 ^B^
PPI4-40	52 ± 6 ^B^	119 ± 10 ^A^	161 ± 2 ^B^	70 ± 1 ^B^

**Table 5:** Least gelation concentration (LGC) of pea protein isolate (PPI) types and pea protein concentrate (PPC). (-) indicates no gelling, (+) indicates gelling occurred, (++) indicates formation of strong gel, and (/) indicates no gelling occurred but an increase in viscosity was noted.

	LGC				
	12%	14%	16%	18%	20%
PPI1	-	-	+	+	++
PPI2	-	/	+	++	++
PPI3	-	/	+	++	++
PPI4	+	+	++	++	++
PPC	+	+	++	++	++

**Table 6:** Differential scanning calorimetry (DSC) of pea protein isolate (PPI) types and pea protein concentrate (PPC). Cells labeled with the same letter are not significant. ^1^ No significant difference was observed for enthalpy. F value: 2.13, *p* > 0.152.

	Onset Temperature (°C)	Peak Denaturation Temperature (°C)	End Temperature (°C)	^1^ Enthalpy (J/g)
PPI1	161.50 ^A^	190.82 ^A^	206.75 ^A^	18.88
PPI2	159.59 ^AB^	183.76 ^B^	199.32 ^B^	19.75
PPI3	153.93 ^C^	181.38 ^BC^	196.04 ^BC^	15.30
PPI4	154.42 ^BC^	180.20 ^C^	192.48 ^BC^	13.75
PPC	156.85 ^ABC^	175.79 ^D^	190.81 ^C^	20.58

**Table 7:** Transverse to longitudinal cutting test ratios for high-moisture meat analog (HMMA) recipes differing in pea protein isolate (PPI) type or PPI1 inclusion level.

Recipe	Firmness Ratio	Toughness Ratio
PPI1-40	1.11	1.16
PPI2-40	1.18	1.10
PPI3-40	1.17	1.14
PPI4-40	1.30	1.29
PPI1-30	1.53	1.69
PPI1-40	1.11	1.16
PPI1-50	1.29	1.20
PPI1-60	0.94	1.13

**Table 8:** Texture and flavor attributes of HMMA products developed by sensory panel for descriptive analyses study.

Attribute	Definition	Reference
Springiness (Texture)	Degree to which sample returns to its original height when compressed once partially with molar teeth and slowly released.	Oscar Mayer Uncured Bun-Length Wieners
Denseness (Texture)	The degree of compactness of the cross section.	Oscar Mayer Uncured Bun-Length Wieners
Juiciness (Texture)	The amount of liquid expressed from the sample at the maximum intensity from 5 chews.	Hormel Cure 81 Ham
Residual Particles (Texture)	Particles remaining in mouth after mastication and swallowing. Maybe fibers, flakes and/or granules.	Hormel Cure 81 Ham
Firmness (Texture)	The force required to bite completely through the meat pieces with the molar teeth.	Hormel Cure 81 Ham
Chewiness (Texture)	Difficulty with which the sample can be broken down with the molars for swallowing.	Hormel Cure 81 Ham
Fiber Awareness (Texture)	The perception of filaments or strands of muscle tissue in product during mastication.	Hillshire Farms Lit’l Beef Smokies
Tooth Packing (Texture)	The amount of sample packed in a between the molar teeth after swallowing.	Wheaties
Astringent (Texture)	A drying puckering or tingling sensation on the surface and/or edge of the tongue and mouth.	Alum Solution
Starchy (Texture)	Degree to which the sample mixes with saliva to form a starchy, pasty slurry that coats mouth surfaces after swallowing.	American Beauty Elbo-Roni
Beany (Flavor)	A slightly brown, musty, slightly nutty, and starchy flavor associated with cooked beans.	Bush’s Best Pinto Beans
Starchy (Flavor)	The dry aromatics associated with starch and starch-based grain products such as wheat, rice, oats, and other grains.	Bush’s Best Pinto Beans
Grain (Flavor)	A general term used to describe the aromatic which includes musty, dusty, slightly brown, slightly sweet and is associated with harvested grains and dry grain stems.	Cereal Mixture (dry)
Green (Flavor)	A green aromatic associated with fresh green peapods. May include beany, increased pungent, musty/earthy, bitter and astringent.	Great Value Frozen Baby Lima Beans
Umami (Flavor)	A general term for aromatics associated with juices from cooked seafood, meat and/or vegetables.	Button Mushroom Broth
Barnyard (Flavor)	Combination of pungent, slightly sour, hay-like aromatics associated with farm animals and the inside of a barn.	White Pepper in water
Cardboard (Flavor)	The flat aromatics that may be associated with cardboard or paper packaging.	Cardboard soaked in water

**Table 9:** Least square (LS) means for texture attributes from sensory analysis of pea-based high-moisture meat analogs (HMMAs) and animal meat anchors. Within each column, values labeled with the same letter are not significant. ^1^ No significant differences were seen for springiness, juiciness, residual particles, fiber awareness, tooth packing, and astringent attributes. F value: 1.89, *p* > 0.116 for springiness. F value: 2.16, *p* > 0.076 for juiciness. F value: 0.62, *p* > 0.683 for residual particles. F value: 2.20, *p* > 0.073 for fiber awareness. F value: 0.19, *p* > 0.963 for tooth packing. F value: 0.94, *p* > 0.465 for astringent.

Recipe	^1^ Springiness	Denseness	Juiciness	Residual Particles	Firmness	Chewiness	Fiber Awareness	Tooth Packing	Astringent	Starchy
PPI3-40	5.19	6.94 ^B^	5.94	4.25	8.94 ^CD^	8.25 ^b^	7.25	2.06	2.81	2.19 ^AB^
PPI4-40	4.31	7.94 ^AB^	5.50	3.81	9.69 ^bc^	9.00 ^ab^	7.19	2.19	2.81	2.69 ^a^
PPI1-30	4.56	8.81 ^ab^	5.00	3.63	10.56 ^b^	9.31 ^ab^	6.88	2.19	2.13	1.00 ^BC^
PPI1-60	3.19	8.31 ^AB^	3.88	3.56	13.00 ^a^	11.13 ^a^	6.75	2.00	3.00	3.38 ^a^
Beef Steak	3.75	11.00 ^a^	6.19	3.56	9.44 ^bcD^	8.63 ^b^	8.31	1.63	2.25	0.25 ^C^
Chicken Breast	3.31	8.94 ^ab^	5.63	3.19	8.25 ^D^	7.75 ^b^	7.88	2.19	1.69	0.25 ^C^

**Table 10:** Least square (LS) means for flavor attributes from sensory analysis of pea-based high-moisture meat analogs (HMMAs) and animal meat anchors. Within each column, values labeled with the same letter are not significant. ^1^ No significant differences were seen for umami and barnyard attributes.

Recipe	Beany	Starchy	Grain	Green	^1^ Umami	Barnyard	Cardboard
PPI3-40	5.06 ^a^	3.13 ^a^	3.44 ^a^	2.81 ^a^	2.13	2.06	2.56 ^a^
PPI4-40	4.88 ^a^	3.00 ^a^	3.69 ^a^	2.63 ^a^	2.31	2.13	3.06 ^a^
PPI1-30	3.69 ^a^	1.69 ^AB^	2.19 ^ab^	1.56 ^ab^	2.00	1.50	1.75 ^ab^
PPI1-60	4.88 ^a^	3.19 ^a^	3.50 ^a^	2.31 ^a^	1.81	2.19	2.81 ^a^
Beef Steak	0.25 ^b^	0.00 ^B^	1.00 ^b^	0.25 ^b^	2.63	0.69	0.50 ^b^
Chicken Breast	0.25 ^b^	0.50 ^B^	0.81 ^b^	0.50 ^b^	2.88	1.13	0.25 ^b^

## Data Availability

The original contributions presented in the study are included in the article, further inquiries can be directed to the corresponding author.

## Practical Application

There are a variety of commercial pea protein isolate types available for purchase on the market but many of these have different functionalities. The specific type and inclusion amount of pea protein isolate has an impact on the processing and final texture of extruded high-moisture meat analogs. Having the ability to relate the inherent functional differences of pea proteins to final product texture will help accelerate the development of fresh, pea-based meat analogs. Addition-ally, increasing the level of pea protein inclusion will increase the texturization in extruded plant-based meat products.

## Abbreviations

HMMA = high-moisture meat analog; GMO = genetically modified organism; PPI = pea protein isolate; RVA = rapid visco anlaysis; SDS-GAGE = sodium dodecyl sulphate–poly- acrylamide gel electrophoresis; LGC = least gelation concentration; TVP = textured vegetable protein; PPC = pea protein concentrate; DI = deionized; SEC-HPLC = size exclusion chromatography by high performance liquid chromatography; DSC = differential scanning calorimeter; SME = specific mechanical energy; IBM = in-barrel moisture; TPA = texture profile analysis; ANOVA = analysis of variance; PCA = principal component analysis.
